# Nowcasting to Monitor Real-Time Mpox Trends During the 2022 Outbreak in New York City: Evaluation Using Reportable Disease Data Stratified by Race or Ethnicity

**DOI:** 10.2196/56495

**Published:** 2025-01-14

**Authors:** Rebecca Rohrer, Allegra Wilson, Jennifer Baumgartner, Nicole Burton, Ray R Ortiz, Alan Dorsinville, Lucretia E Jones, Sharon K Greene

**Affiliations:** 1 Bureau of Communicable Disease, New York City Department of Health and Mental Hygiene, Long Island City, NY, United States

**Keywords:** data quality, epidemiology, forecasting, infectious disease, morbidity and mortality trends, mpox, nowcasting, public health practice, surveillance

## Abstract

**Background:**

Applying nowcasting methods to partially accrued reportable disease data can help policymakers interpret recent epidemic trends despite data lags and quickly identify and remediate health inequities. During the 2022 mpox outbreak in New York City, we applied Nowcasting by Bayesian Smoothing (NobBS) to estimate recent cases, citywide and stratified by race or ethnicity (Black or African American, Hispanic or Latino, and White). However, in real time, it was unclear if the estimates were accurate.

**Objective:**

We evaluated the accuracy of estimated mpox case counts across a range of NobBS implementation options.

**Methods:**

We evaluated NobBS performance for New York City residents with a confirmed or probable mpox diagnosis or illness onset from July 8 through September 30, 2022, as compared with fully accrued cases. We used the exponentiated average log score (average score) to compare moving window lengths, stratifying or not by race or ethnicity, diagnosis and onset dates, and daily and weekly aggregation.

**Results:**

During the study period, 3305 New York City residents were diagnosed with mpox (median 4, IQR 3-5 days from diagnosis to diagnosis report). Of these, 812 (25%) had missing onset dates, and of these, 230 (28%) had unknown race or ethnicity. The median lag in days from onset to onset report was 10 (IQR 7-14). For daily hindcasts by diagnosis date, the average score was 0.27 for the 14-day moving window used in real time. Average scores improved (increased) with longer moving windows (maximum: 0.47 for 49-day window). Stratifying by race or ethnicity improved performance, with an overall average score of 0.38 for the 14-day moving window (maximum: 0.57 for 49 day-window). Hindcasts for White patients performed best, with average scores of 0.45 for the 14-day window and 0.75 for the 49-day window. For unstratified, daily hindcasts by onset date, the average score ranged from 0.16 for the 42-day window to 0.30 for the 14-day window. Performance was not improved by weekly aggregation. Hindcasts underestimated diagnoses in early August after the epidemic peaked, then overestimated diagnoses in late August as the epidemic waned. Estimates were most accurate during September when cases were low and stable.

**Conclusions:**

Performance was better when hindcasting by diagnosis date than by onset date, consistent with shorter lags and higher completeness for diagnoses. For daily hindcasts by diagnosis date, longer moving windows performed better, but direct comparisons are limited because longer windows could only be assessed after case counts in this outbreak had stabilized. Stratification by race or ethnicity improved performance and identified differences in epidemic trends across patient groups. Contributors to differences in performance across strata might include differences in case volume, epidemic trends, delay distributions, and interview success rates. Health departments need reliable nowcasting and rapid evaluation tools, particularly to promote health equity by ensuring accurate estimates within all strata.

## Introduction

### Timeline and Motivation

In 2022, an mpox outbreak occurred in countries where local transmission previously had not been observed, including the United States [1]. New York City was the first urban center in the United States to experience a rapid increase in cases [2]. The first case among New York City residents was diagnosed on May 19, 2022 [3]. The next day, New York City health care providers were notified to immediately report suspected cases to the Provider Access Line at the New York City Department of Health and Mental Hygiene (New York City Health Department) for potential testing through the Public Health Laboratory [3]. On June 21, 2022, the New York City Health Department Incident Command System was activated for a public health response, and on July 8, the New York State Department of Health notified health care providers of the availability of commercial laboratory testing for mpox [4]. The New York City Health Department declared a local state of emergency on August 1 [5], and the Secretary of Health and Human Services declared a nationwide public health emergency on August 4 [6]. As the outbreak subsided, the New York City Health Department partially deactivated mpox emergency response activities on October 31 and fully deactivated these activities on January 31, 2023, aligning with the expiration of the US public health emergency declaration [7].

Throughout the emergency response, the New York City Health Department tracked case counts internally and on a public-facing website [8]. Inherent delays (eg, from patient symptom onset to care seeking, laboratory testing, provider and laboratory reporting to the New York City Health Department, and phone interviews with patients to determine the date of onset) make it difficult to interpret recent epidemic trends and make timely decisions during an outbreak. In early August 2022, while reviewing daily epidemic curves with no accounting for data lags, the New York City Health Department leadership inquired whether the outbreak had peaked.

### Health Inequities Across Race or Ethnicity Groups

**Figure 1.**
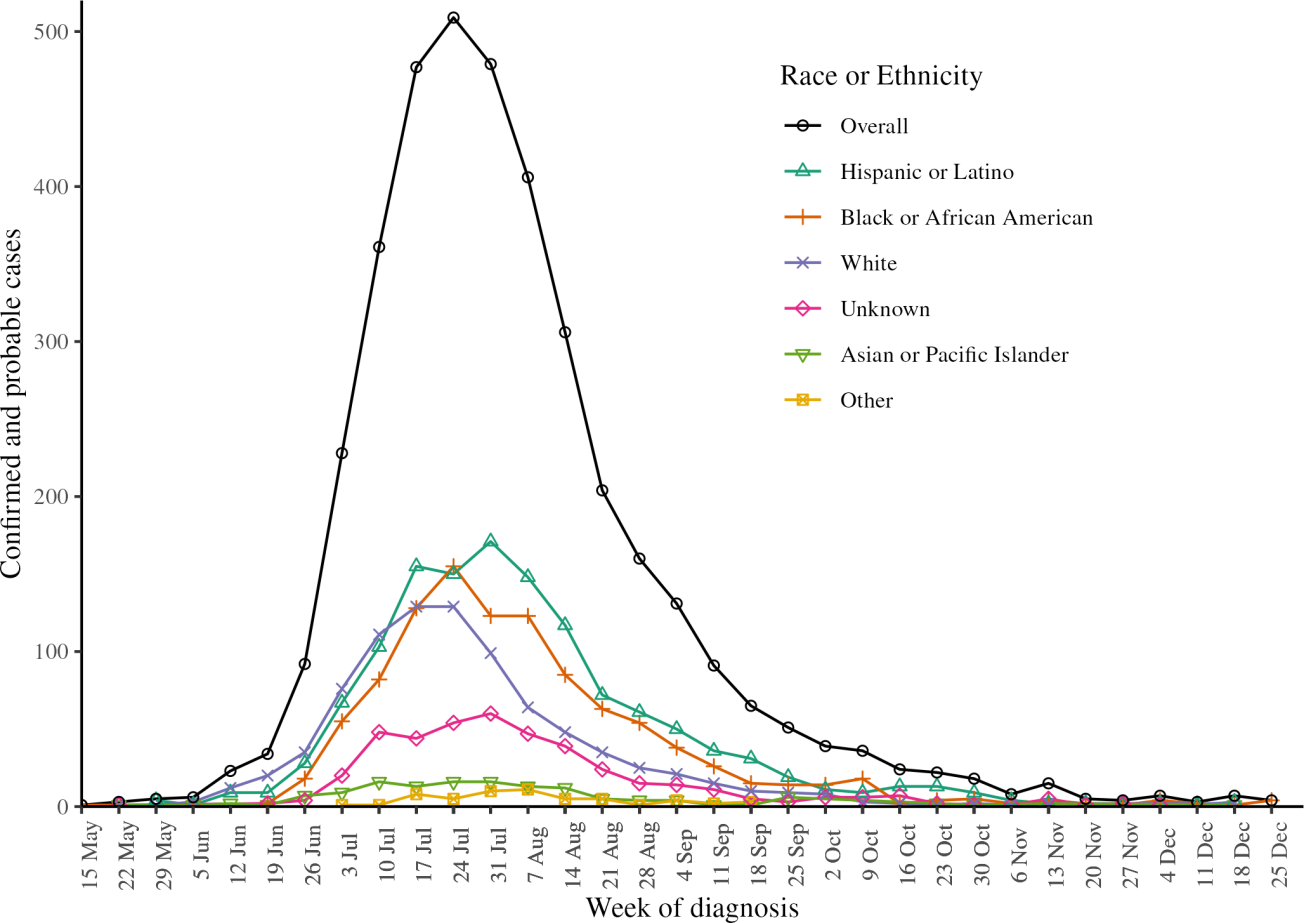
Weekly confirmed and probable mpox cases among New York City residents diagnosed from May through December 2022, overall and stratified by race or ethnicity.

The burden of mpox diagnoses was inequitably distributed by race and ethnicity among patients in the United States [9] and in New York City [10]. Confirmed and probable mpox diagnoses [11] among New York City residents peaked first among White individuals (weeks beginning July 17 and July 24, 2022), then among Black or African American individuals (week beginning July 24, 2022), and then among Hispanic or Latino individuals (week beginning July 31, 2022; Figure 1). Cases then decreased most sharply first among White individuals, then among Black or African American individuals, and then among Hispanic or Latino individuals. Differences in the timing, magnitude, and duration of epidemic peaks by race or ethnicity could reflect, in part, true epidemiologic differences, such as sexual network effects including exposures while traveling early in the outbreak, before local transmission was established, and differences in access to vaccination and treatment [12-16]. In addition, systemic inequities, including heightened stigma, medical mistrust, and inaccessibility of health care services (including financial barriers, inadequate insurance coverage, not having access to a primary care provider, lack of transportation, and lack of convenient care locations) likely contributed to reduced or delayed case ascertainment among Black or African American and Hispanic or Latino individuals [16-21]. Additionally, public health messaging and outreach did not quickly and effectively reach all affected persons, due in part to insufficient accommodation for cultural nuances and linguistic diversity, further contributing to care-seeking delays [17,19,22].

### Nowcasting and the COVID-19 Pandemic Precedent

“Nowcasting” refers to predicting the present, and “hindcasting” refers to predicting through the day before the present. Nowcasting and hindcasting methods can be applied to partially accrued reportable disease data to estimate the number of recent events that have not yet been reported [23,24]. Public health agencies have nowcasted various infectious diseases [24-26].

The New York City Health Department first used nowcasting to improve real-time situational awareness during the COVID-19 pandemic public health emergency [24], applying a method called Nowcasting by Bayesian Smoothing (NobBS) [23,27]. NobBS requires a case line list of “date of interest” and “report date” to assess the past delay distribution and epidemic trend and projects the number of cases during a user-specified moving window ending on a date representing “now” [23].

We applied lessons learned from an evaluation of nowcasting COVID-19 [24] to mpox, including (1) using a negative binomial distribution instead of the NobBS default Poisson distribution, (2) using a 2-week moving window length for diagnoses, and (3) removing the display of estimates of diagnoses on weekends, given lack of adjustment for day-of-week effects. Additionally, we wished to nowcast mpox by onset date and to stratify by race or ethnicity, neither of which was previously implemented for COVID-19 at the New York City Health Department. We sent daily automated nowcasting reports to surveillance data leadership starting September 19, 2022; implementation delays were driven by complexities in determining the onset report date and limited staff resources. To monitor differences in epidemic trends across groups, we started stratifying nowcasts by race or ethnicity on September 29.

### Objectives

First, we documented challenges in developing input files for daily hindcasts of mpox cases among New York City residents by diagnosis date and by onset date, overall and stratified by race or ethnicity. Our goal was to provide methodologists developing nowcasting tools with greater insight into how relevant data are collected locally during a public health emergency. Second, we conducted a retrospective evaluation of hindcasting performance for New York City residents diagnosed with confirmed or probable mpox [11] from July 8 through September 30, 2022, capturing the outbreak peak and decline, compared with fully accrued case counts as of September 1, 2023. We used a 14-day moving window for hindcasting by diagnosis date and a 21-day moving window for hindcasting by onset date in real time and assessed whether other moving window lengths or a weekly time unit would have performed better. Third, we assessed mpox hindcast accuracy when stratifying by race or ethnicity.

## Methods

### Data Collection

We used onset, diagnosis, and reporting dates, as well as race and ethnicity data from the New York City Health Department’s mpox surveillance database. Reports were imported electronically from laboratories via the New York State Electronic Clinical Laboratory Reporting System [28,29] and from health care providers via Reporting Central, through the electronic Universal Reporting Form [30]. Information from providers reporting by phone was entered into the surveillance database via on-call physician notes. We included patients who tested positive for either mpox virus (confirmed cases) or orthopoxvirus (probable cases), as detailed in standard case definitions [11].

The Surveillance and Investigations Unit of the Mpox Emergency Response Team at the New York City Health Department conducted patient phone interviews as soon as possible after the initial report of diagnosis to determine risk factors for exposure, identify contacts, and prevent further transmission. These interviews included questions on symptom onset date, self-reported race and ethnicity, and recent history of sexual contact. Responses were entered into the surveillance database.

### Data Point Selection

We selected the relevant “dates of interest” (diagnosis or onset date) and their respective report dates. The diagnosis date was defined as the specimen collection date of the first positive laboratory test, which was ascertained via electronic laboratory reporting. The symptom onset date for mpox illness was elicited during the patient interview and manually entered. The respective report dates were the different dates that the New York City Health Department ascertained the dates of interest. The diagnosis report date was defined as the date the first positive laboratory result indicating a patient met confirmed or probable case criteria [11] was received by the New York City Health Department. The onset report date was calculated based on the source for establishing the onset date, which was most commonly patient interview (Table 1).

We reviewed cases with long (≥50 days) or negative spans between date of interest and its report date to identify cases requiring additional data cleaning. Patients with a missing onset date were excluded from onset nowcasting. Of 2493 patients diagnosed during the study period and with an available onset date, 2099 (84%) had different report dates for diagnosis and onset, with a median of 2 (IQR 1‐4) days between diagnosis report date and onset report date.

**Table 1.** Mpox onset report date sources, in descending order of preference, as available from the New York City Health Department’s surveillance database and as used for Nowcasting by Bayesian Smoothing.

Onset date source	Onset report date source	Onset report date source for 2278 patients with an available onset date from July 8 through September 30, 2022, n (%)
Health care provider report, where onset date on form matches mpox onset date in case record	Electronic universal reporting form receipt date	35 (1.5)
Patient interview	Interview date	2038 (89.5)
Administrative log	Date administrative interview log was changed for the final time from “Assigned” to another status, for example, “Complete” or “Sent to supervisor for review”^a^	23 (1.0)
Any source, if onset before August 1 or outlier in quality assurance review	Manually hard-coded based on free-text notes in the surveillance database	49 (2.2)
Any source, if no other date available, or if later than the date set earlier in the hierarchy	Date the case was first set as confirmed or probable	133 (5.8)

a Applied to patients with onset starting August 1, 2022. Before then, interview dates were likely to be reported in on-call physician notes only, and assigning the onset report date based on the interview log would have been inaccurate.

On September 1, 2023, we created a frozen analytic line list of mpox cases among New York City residents with the minimum necessary variables to evaluate nowcasting performance—diagnosis date, diagnosis report date, onset date, onset report date, and race or ethnicity. This dataset was separately filtered by patients with diagnosis (n=3305) or known illness onset (n=2278) during the study period, from July 8 through September 30, 2022. We started the study period on July 8, 2022, when commercial laboratory testing became available, and ended on September 30, 2022, because case counts were sparse thereafter (Figure 1).

We characterized the delay distribution from diagnosis to diagnosis report and from onset to onset report by median number of days, IQR, and 90th percentile. We assessed delays overall during the study period and stratified by month and race or ethnicity. We used Kruskal-Wallis tests to assess whether delay distributions varied across race or ethnicity.

### Retrospective Nowcasting Evaluation

We mimicked prospective surveillance on Wednesdays for case counts through Tuesdays by using the R package NobBS (The R Foundation) [27] and restricting to data that had been available at the time. We evaluated hindcast performance across moving window length (14, 21, 28, 35, 42, and 49 days and 2, 3, 4, 5, 6, and 7 weeks), time unit (day vs week), and stratification (overall or stratified by race or ethnicity). For the maximum delay value, we used the NobBS default of the moving window length minus 1.

We chose to mimic surveillance on Wednesdays to balance operational constraints. Hindcast estimates produced on Mondays and Tuesdays could be underestimated because of reduced care-seeking and laboratory reporting on weekends, and hindcasts conducted on Thursdays and Fridays might be received by decision makers too late in the work week to affect that week’s planned public health actions.

To evaluate moving window lengths at daily resolution, we retained the number of estimated cases for each of the prior 7 days. For weekly resolution, we aggregated cases to 7-day periods and retained the estimate for the most recent week. While data from diagnoses on all days of the week were included in model inputs, when conducting the performance evaluation, we evaluated only daily diagnosis estimates from weekdays. This was because diagnoses were reduced on weekends when health care provider availability was more limited. This exclusion did not apply to estimates of onsets or weekly time periods.

Each window length was assessed for periods ending on Tuesdays once the number of days or weeks of that window length had elapsed since the July 8, 2022, start date. For example, we assessed the performance of a 14-day moving window beginning the 14-day period from July 13 through 26, 2022, shifting forward 1 week from July 20 through August 2, and continuing to shift forward 1 week at a time until ending with the period from September 14 through 27, 2022, for a total of 10 models run. For each model, we retained diagnoses for the last 7 days in the window, then excluded weekends, for a total of 50 estimates (5 weekdays from each of 10 models with different end dates). These 50 estimates were used for the performance evaluation. Scenarios with longer moving windows or with weekly aggregation had fewer estimates available for evaluation.

When stratifying by race or ethnicity, we used the “strata” option in NobBS. This option estimated the delay distribution across all race and ethnicity groups and the epidemic curves separately for each group. These analyses were restricted to Black (including African American or Afro-Caribbean), Hispanic or Latino, and White patients because of low case counts in other groups, including Asian, Native Hawaiian or Pacific Islander, and Native American or Alaska Native. We suspected the delay distribution could vary across race or ethnicity groups given differential access to diagnosis and accessibility for interviews, motivating us to compare the accuracy of stratified and unstratified estimates.

For each date of interest (ie, diagnosis or onset), we evaluated groups of estimates—moving window lengths against the lengths used in real time, stratified estimates, and weekly versus daily time units. Drawing from prior evaluations, we evaluated hindcasting performance using the log score [23], mean absolute error (MAE) [24,31,32], relative root mean square error (rRMSE) [24], and 95% prediction interval (PI) coverage [24,32].

We used the log score to evaluate the accuracy of the posterior predictive distribution of each hindcast [23]. We assigned predictive distributions to bins of possible values of fully accrued case counts. For unstratified hindcasts, we used bin widths of 10 cases ranging from 0‐99 for daily hindcasts and of 50 cases ranging from 0‐549 for weekly hindcasts. For stratified hindcasts, we used bin widths of 5 cases ranging from 0‐39 for daily hindcasts and of 20 cases ranging from 0‐179 for weekly hindcasts. These bin widths were selected to yield similar numbers of bins (10, 11, 8, or 9 bins, respectively), to enable comparisons across scenarios with widely varying case volumes. The log score was the natural log of the probability assigned to the bin in which the true count fell [23]. If the probability assigned to the bin for the true count was 0, then we assigned a lower limit log score of −10; this was necessary for only one estimate, for hindcasting for August 23, 2022, by week of onset using a 4-week moving window, stratified among Hispanic or Latino patients. We calculated the average log score across all days or weeks retained for evaluation. We report the exponentiated average log score (ie, average score), which is the average probability NobBS assigned to the bin containing the true case count [23]. Higher average scores indicated more accurate performance.

We also calculated the daily or weekly MAE and average daily or weekly rRMSE across all individual days or weeks evaluated to compare point estimates of hindcasted cases with the final number of cases reported after data accrued. Lower MAE and lower rRMSE indicated better performance, with estimates closer to final counts. MAE is dependent on case volume, making it useful for comparing scenarios with similar case volumes, such as the same time unit and stratification. rRMSE was more useful than MAE for comparing scenarios with different case volumes, which allowed us to compare daily versus weekly and stratified versus unstratified estimates. The 95% PI coverage represents the percentage of estimates when the 95% PI included the final case count; the closer to 95%, the better the performance is. When the 95% PI coverage is near 100%, then PIs might be too wide to be informative.

We checked the dispersion ratio for the entire study period and for shorter periods of 14- and 21-day duration ending on Tuesdays to reflect the window lengths used in real time for diagnosis and onset. This was done using Poisson regression models of counts by each respective date to confirm whether a negative binomial data distribution was appropriate for this dataset.

### Ethical Considerations

The New York City Health Department’s institutional review board reviewed this work and determined it to be exempt human participants research under 45 CFR §46.104(d)(4)(ii) and (iii) (IRB No. 22‐097). Analyses were performed using R version 4.2 and NobBS version 0.1.0. The frozen analytic line list, evaluation code, and codebook are available on GitHub [33].

## Results

### Data Lags and Interview Completeness

Among 3305 New York City residents diagnosed with mpox from July 8 through September 30, 2022, the median lag in days from diagnosis to diagnosis report was 4 (IQR 3-5, 90th percentile: 6). Lags decreased as the epidemic progressed, from a median lag of 4 days for patients diagnosed in July to 3 days for those diagnosed in September (Table 2). Of 3305 patients diagnosed with mpox, 2558 (77%) were probable cases, with a median lag in days from diagnosis to diagnosis report of 4 (IQR 3‐5, 90th percentile: 6). The remaining 747 (23%) were confirmed cases, with a shorter median lag of 3 (IQR 2‐4, 90th percentile: 5) days (Table 2). Of the 3305 diagnosed patients, 2429 (73%) had a fully or partially completed interview (Table S1 in Multimedia Appendix 1). Typically, the interview was conducted within a median of 1 (IQR 1‐4) day of when the Health Department was notified of a confirmed or probable case, and a median of 10 (IQR 7‐14) days of disease onset. The interview success rate was steady by diagnosis week, with a weekly median of 73% of patients successfully interviewed (range 64%‐80%).

**Table 2.** Lags from diagnosis to diagnosis report and from onset to onset report among New York City residents with confirmed or probable mpox diagnosis or onset from July 8 through September 30, 2022, by case status and month.

Date of interest, period, and stratification			Median number of days from date of interest to report of date of interest (IQR), 90th percentile			Values, n			*P* value^a^ for Kruskal-Wallis test across race or ethnicity strata		
			Confirmed + Probable	Confirmed	Probable	Confirmed + Probable	Confirmed	Probable	Confirmed + Probable	Confirmed	Probable
**Diagnosis**											
	**July 8-September 30**								.75	.47	.54
		Unstratified	4 (3-5), 6	3 (2-4), 5	4 (3-5), 6	3305	747	2558	—^b^	—	—
		Black or African American	4 (3-5), 6	3 (2-4), 5	4 (3-5), 6	919	231	688			
		Hispanic or Latino	4 (3-5), 6	3 (2-4), 5	4 (3-5), 6	1131	257	874			
		White	4 (3-5), 6	3 (3-4), 5	4 (3-5), 6	716	118	598			
	**July 8‐31**								.33	.34	.39
		Unstratified	4 (3-5), 6	4 (3-4), 5	4 (3-5), 6	1458	101	1357	—	—	—
		Black or African American	4 (3-5), 6	4 (3-5), 5	4 (3-5), 6	388	29	359			
		Hispanic or Latino	4 (3-5), 6	3 (3-4), 5	4 (3-5), 6	448	27	421			
		White	4 (3-5), 6	4 (3-5), 5	4 (3-5), 6	395	22	373			
	**August 1‐31**								.93	>.99	.76
		Unstratified	4 (3-5), 7	3 (2-4), 5	4 (3-6), 7	1463	472	991	—	—	—
		Black or African American	4 (3-5), 7	3 (2-5), 5	4 (3-6), 7	421	153	268			
		Hispanic or Latino	4 (3-5), 6	3 (2-4), 6	4 (3-6), 6	528	157	371			
		White	4 (3-5), 7	3 (2-4), 5	4 (3-6), 8	262	72	190			
	**September 1‐30**								.60	.35	.98
		Unstratified	3 (2-3), 4	3 (1-3), 4	3 (2-4), 4	384	174	210	—	—	—
		Black or African American	3 (2-3), 4	3 (1-3), 4	3 (2-4), 4	110	49	61			
		Hispanic or Latino	3 (2-3), 4	3 (1-3), 4	3 (2-3), 4	155	73	82			
		White	3 (2-4), 5	3 (2-4), 4	3 (2-4), 5	59	24	35			
**Onset**											
	**July 8-September 30**								.97	.74	.97
		Unstratified	10 (7‐14), 18	9 (6-13), 18	10 (8‐14), 18	2278	542	1736	—	—	—
		Black or African American	10 (7‐14), 18	9 (6-12), 17	10 (8‐14), 18	648	174	474			
		Hispanic or Latino	10 (7‐14), 18	8 (7-13), 20	10 (7‐14), 18	876	215	661			
		White	10 (8‐13), 17	9 (6-12), 17	10 (8‐13), 18	501	94	407			
	**July 8‐31**								.21	.66	.09
		Unstratified	11 (9‐15), 19	11 (7‐17), 21	11 (9‐14), 19	1185	100	1085	—	—	—
		Black or African American	11 (9‐15), 19	11 (7‐14), 18	11 (9‐15), 19	324	28	296			
		Hispanic or Latino	11 (9‐15), 20	9 (7-17), 21	11 (9‐15), 20	412	35	377			
		White	11 (9‐14), 18	11 (9‐16), 29	11 (9‐13), 18	311	21	290			
	**August 1‐31**								.72	.30	.93
		Unstratified	9 (6-12), 16	8 (6-12), 18	9 (6-12), 15	866	338	528	—	—	—
		Black or African American	9 (6-12), 16	9 (6-13), 18	9 (6-12), 15	254	116	138			
		Hispanic or Latino	9 (6-12), 16	9 (7-13), 20	9 (6-12), 16	363	132	231			
		White	9 (6-12), 14	8 (5-11), 16	9 (7-12), 14	158	59	99			
	**September 1‐30**								.79	.87	.74
		Unstratified	8 (6-10), 14	8 (5-10), 14	8 (6-11), 14	227	104	123	—	—	—
		Black or African American	8 (5-10), 14	8 (5-11), 14	7 (5-10), 15	70	30	40			
		Hispanic or Latino	8 (6-10), 14	8 (6-10), 14	8 (6-11), 14	101	48	53			
		White	8 (6-11), 14	8 (5-9), 13	8 (6-12), 20	32	14	18			

a *P* values were unadjusted for multiple comparisons.

b Not applicable. Em dashes indicate there was no statistical test performed for unstratified values.

Of patients who were not interviewed, 88% (n=767) had missing onset dates and 28% (n=248) had unknown race or ethnicity (Table S1 in Multimedia Appendix 1). Race or ethnicity distributions were similar between patients who were and were not interviewed, except 39% (n=943) of interviewed patients were Hispanic or Latino, compared with only 21% (n=188) of not interviewed patients (Table S1 in Multimedia Appendix 1). The lower interview success rate among Hispanic or Latino patients could have reduced hindcasting performance for this stratum.

Separately, during the study period, 2278 patients had a recorded mpox illness onset date, and the median lag in days from onset to onset report was 10 (IQR 7‐14, 90th percentile: 18). Lags decreased from a median of 11 days for patients with onset in July to 8 days in September (Table 2). Of 2278 patients with an illness onset date, 1736 (76%) were probable cases, with a median lag from onset to onset report of 10 (IQR 8‐14, 90th percentile: 18) days. The remaining 542 (24%) were confirmed cases, with a shorter median lag of 9 (IQR 6‐13, 90th percentile: 18) days (Table 2).

There was no statistically significant difference at α=.05 across race or ethnicity groups in the lag from diagnosis to diagnosis report or the lag from onset to onset report, overall or in any individual month based on the results of Kruskal-Wallis tests (Table 2). Of 2278 patients with an onset date, 53 (2%) purportedly had onset after diagnosis, representing recall or data entry quality issues. Of the remaining 2225, the median lag in days from onset to diagnosis was 4 (IQR 2‐7, 90th percentile: 10) (Table S2 in Multimedia Appendix 1).

Of 3305 patients diagnosed during this period, 812 (25%) were missing onset date (Table 3). Of these, 230 (28%) also had unknown race or ethnicity (Table 3). Onset date missingness increased with time, from 19% (n=278) for patients diagnosed in July to 31% (n=117) for those diagnosed in September (Table 4).

Counts of cases for the full study period and for 14-day windows by diagnosis date were consistently overdispersed in Poisson regression models by diagnosis date and less so for 21-day windows by onset date (Table S3 in Multimedia Appendix 1), supporting use in NobBS of a negative binomial case distribution.

**Table 3.** New York City residents diagnosed with mpox from July 8 through September 30, 2022, by onset date missingness, race or ethnicity, and interview status.

Patient characteristic	Missing onset date (n=812), n (column %)	Total (n=3305), n (column %)
**Race or ethnicity**		
Asian or Pacific Islander	20 (2.5)	109 (3.3)
Black or African American	221 (27.2)	919 (27.8)
Hispanic or Latino	182 (22.4)	1131 (34.2)
White	151 (18.6)	716 (21.7)
Other	8 (1.0)	56 (1.7)
Unknown	230 (28.3)	374 (11.3)
**Interviewed**		
Yes	45 (5.5)	2429 (73.5)
No	767 (94.5)	876 (26.5)

**Table 4.** New York City residents diagnosed with mpox from July 8 through September 30, 2022, by onset date missingness and diagnosis month.

Diagnosis month	Missing onset date, n (row %)	Total, n
July	278 (19.1)	1458
August	417 (28.5)	1463
September	117 (30.5)	384
Total	812 (24.6)	3305

### Scenario Performance

#### Moving Window Lengths

For daily hindcasting unstratified by race or ethnicity, both by diagnosis and onset date, no single scenario performed best across MAE, rRMSE, 95% PI coverage, and average score (Table 5, Table S4 in Multimedia Appendix 1). For hindcasting by diagnosis date, as moving window lengths increased, the average score generally improved (increased), MAE generally improved (decreased), and rRMSE worsened (increased). Patterns were inconsistent for hindcasting by onset date.

For hindcasting by diagnosis date, the average score for the 14-day moving window used in real time was 0.27, with other scenarios ranging from 0.27 to 0.47 (Table 5). The MAE for the 14-day moving window was 9, with other scenarios ranging from 3 to 9. The rRMSE for the 14-day window was 0.23, with other scenarios ranging from 0.25 to 0.30. The 95% PI coverage for the 14-day window was 96%, with other scenarios ranging from 93% to 100%.

**Table 5.** Performance measures for diagnosis date–based hindcasting approaches in Nowcasting by Bayesian Smoothing, applied to daily case counts of New York City residents with mpox diagnosis from July 13 through September 27, 2022 (metrics calculated on last 7 days of hindcast, excluding weekends).

Stratification and scenario number	Window length (days)^a^, n	Mean absolute error	Relative root mean square error	Number of estimates when the 95% prediction interval included the final case count (95% prediction interval coverage)	Number of estimates evaluated (number of models run)	Average score
**Unstratified**						
1^b^	14	9.04	0.23	48 (96.00)	50 (10)	0.27
2	21	8.73	0.25	42 (93.33)	45 (9)	0.28
3	28	7.18	0.25	37 (92.50)	40 (8)	0.27
4	35	5.09	0.27	35 (100.00)	35 (7)	0.41
5	42	3.93	0.29	29 (96.67)	30 (6)	0.44
6	49	2.88	0.30	24 (96.00)	25 (5)	0.47
**Black or African American**						
7^b^	14	2.90	0.30	49 (98.00)	50 (10)	0.39
8	21	2.16	0.32	44 (97.78)	45 (9)	0.41
9	28	1.77	0.33	39 (97.50)	40 (8)	0.41
10	35	1.43	0.37	35 (100.00)	35 (7)	0.48
11	42	1.10	0.41	30 (100.00)	30 (6)	0.49
12	49	1.24	0.51	25 (100.00)	25 (5)	0.49
**Hispanic or Latino**						
13^b^	14	3.42	0.34	46 (92.00)	50 (10)	0.32
14	21	3.09	0.35	42 (93.33)	45 (9)	0.33
15	28	2.70	0.39	37 (92.50)	40 (8)	0.33
16	35	1.69	0.37	35 (100.00)	35 (7)	0.49
17	42	1.60	0.48	30 (100.00)	30 (6)	0.50
18	49	1.28	0.50	25 (100.00)	25 (5)	0.52
**White**						
19^b^	14	2.10	0.32	48 (96.00)	50 (10)	0.45
20	21	1.69	0.39	44 (97.78)	45 (9)	0.52
21	28	1.38	0.41	39 (97.50)	40 (8)	0.54
22	35	1.20	0.46	35 (100.00)	35 (7)	0.64
23	42	1.10	0.49	30 (100.00)	30 (6)	0.70
24	49	0.76	0.49	25 (100.00)	25 (5)	0.75
**All stratified**						
25^b^	14	2.81	0.32	143 (95.33)	150 (10)	0.38
26	21	2.31	0.35	130 (96.30)	135 (9)	0.41
27	28	1.95	0.38	115 (95.83)	120 (8)	0.42
28	35	1.44	0.40	105 (100.00)	105 (7)	0.53
29	42	1.27	0.46	90 (100.00)	90 (6)	0.55
30	49	1.09	0.50	75 (100.00)	75 (5)	0.57

a 14-, 21-, 28-, 35-, 42-, and 49-day nowcasts started on July 26, August 2, August 9, August 16, August 23, and August 30, 2022, respectively, to provide 2, 3, 4, 5, 6, or 7 weeks of Wednesday-Tuesday data since study start date July 8, 2022. We mimicked nowcasts weekly, ending September 27, 2022, as the last Tuesday during the study period.

b Indicates scenario applied in real time at the New York City Health Department.

For hindcasting by onset date, the average score for the 21-day moving window used in real time was 0.23, with other scenarios ranging from 0.16 for the 42-day window to 0.30 for the 14-day window (Table S4 in Multimedia Appendix 1). The MAE for the 21-day moving window was 12, with other scenarios ranging from 7 to 11. The rRMSE for the 21-day window was 1.07, with other scenarios ranging from 0.75 to 1.42. The 95% PI coverage for the 21-day window was 84%, with other windows ranging from 75% to 99% (Table S4 in Multimedia Appendix 1).

**Figure 2.**
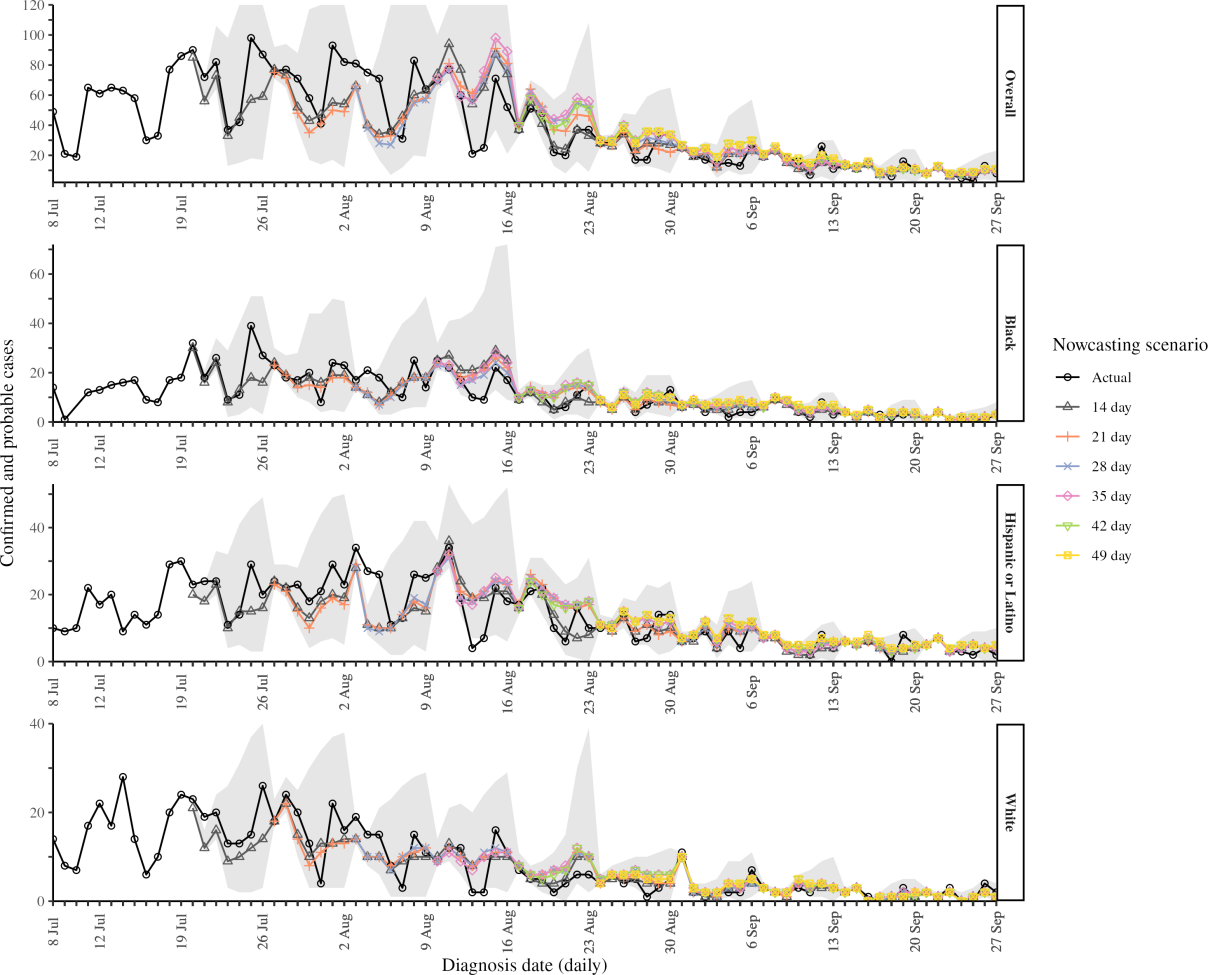
Comparison of 7-day hindcasts conducted on Wednesdays using various moving window lengths at the daily time unit for confirmed and probable mpox cases among New York City residents diagnosed from July 8 through September 27, 2022, overall and stratified by 3 race or ethnicity groups. Final case counts reported as of September 1, 2023, are shown in black. The 95% prediction interval is shown in gray for the 14-day window, which was the scenario implemented in real time. The y-axis for overall diagnoses was truncated at 120 for clarity, but the observed upper bound of the 95% prediction interval for the 14-day window was 252 on August 16, 2022.

Overall, hindcasts underestimated diagnoses in early August 2022, on the downslope of the epidemic curve, then overestimated diagnoses in late August (Figure 2 and Figure S1 in Multimedia Appendix 1). Hindcasting overestimated onsets throughout the study period, except for the 14-day daily and 2-week weekly moving windows, which underestimated cases at points in early and late August 2022 (Figures S2 and S3 in Multimedia Appendix 1). Lags from onset to onset report decreased rapidly in July and August (Figure S4 in Multimedia Appendix 1); the shortening delay distribution over time might have led NobBS to overestimate onsets. By September 2022, diagnoses and onsets were low and stable, and both daily and weekly hindcast estimates, regardless of window length, were close to final diagnosis counts (Figure 2 and Figures S1-S3 in Multimedia Appendix 1).

#### Stratification

For daily diagnosis hindcasts stratified by race or ethnicity, the average score for the 14-day moving window used in real time was 0.38, with other scenarios ranging from 0.41 to 0.57 (Table 5). The average score was higher in stratified estimates compared with unstratified estimates. When evaluating race or ethnicity strata individually, hindcasts for White patients had the highest performance (higher average scores ranging from 0.45-0.75), while hindcasts for Black or African American and Hispanic or Latino patients had lower performance (ranging from 0.39-0.49 and 0.32-0.52, respectively). Worse performance in particular strata could be explained by sparser counts and epidemic trends that are difficult to estimate or by minor differences in the delay distribution and interview success rates across strata.

The rRMSE for the 14-day moving window was 0.32, with other scenarios ranging from 0.35 to 0.50 (Table 5). The 95% PI coverage for the stratified 14-day diagnosis window was 95%, with other scenarios ranging from 96% to 100%. For stratified daily onset hindcasts, the average score for the 21-day window used in real time was 0.36, with other scenarios ranging from 0.36 to 0.54. The rRMSE for the 21-day window was 1.22; others ranged from 0.91 to 1.71 (Table S4 in Multimedia Appendix 1). The 95% PI coverage for the stratified 21-day onset window was 95%; others ranged from 89% to 97%. For any given moving window length, rRMSE increased (worsened) for stratified compared with unstratified estimates in both diagnosis and onset-based hindcasts. For any given moving window length, the 95% PI coverage was not consistently closer to 95% in either the stratified or unstratified scenario.

#### Weekly Time Unit

For unstratified weekly diagnosis hindcasts, the average score remained stable at different window lengths, ranging from 0.25 to 0.30 (Table S5 in Multimedia Appendix 1). This was comparable to the performance of unstratified daily diagnosis hindcasts in shorter window lengths (14, 21, and 28 days) and worse in longer window lengths (35, 42, and 49 days; Table 5). The rRMSE for unstratified weekly diagnosis hindcasts ranged from 0.21 to 0.37 (Table S5 in Multimedia Appendix 1). This was similar to the rRMSE for daily unstratified diagnosis hindcasts, which ranged from 0.23 through 0.30 across moving windows (Table 5). The 95% PI coverage ranged from 83% to 100% (Table S5 in Multimedia Appendix 1).

For unstratified weekly onset hindcasts, the average score was poor across all moving window lengths, ranging from 0.09 to 0.18 (Table S5 in Multimedia Appendix 1), and was worse than the average scores at daily resolution (Table S4 in Multimedia Appendix 1). The rRMSE ranged from 0.24 to 1.10 (Table S5 in Multimedia Appendix 1). This was similar to rRMSE in unstratified daily onset hindcasts, which ranged from 0.75 to 1.42 (Table S4 in Multimedia Appendix 1). The 95% PI coverage ranged from 60% to 100% (Table S5 in Multimedia Appendix 1). For a given moving window length, rRMSE typically increased (worsened) weekly compared with daily diagnosis hindcasts but decreased (improved) weekly compared with daily onset hindcasts. Weekly hindcasts generally had worse 95% PI coverage than their daily counterpart. The lowest performing window length based on 95% PI coverage was much worse for unstratified weekly scenarios (83% for diagnosis and 60% for onset; Table S5 in Multimedia Appendix 1) than for daily scenarios (93% for diagnosis and 75% for onset; Table 5, Table S4 in Multimedia Appendix 1).

## Discussion

### Principal Findings

In evaluating NobBS for the 2022 mpox outbreak in New York City, we faced challenges in developing input files using the onset date. In addition, no moving window length consistently performed best. Daily time units performed better than weekly, and stratifying by race or ethnicity improved performance.

A key challenge in developing input files was that the onset date was frequently missing, which is a common challenge for mpox data collected via patient interviews [34]. When the onset date was available, it was usually after a long delay; the 90th percentile of delay from onset to onset report was 18 days (Table 2), reducing the usefulness of shorter moving window lengths. Furthermore, the onset report date was not a standardized field in our disease surveillance database, which led to implementation delays during the public health emergency. Performance was better when hindcasting by diagnosis date than by onset date, as expected given shorter lags from diagnosis to diagnosis report than from onset to onset report and missingness in onset date.

The choice of moving window length and whether to stratify by race or ethnicity had less influence on hindcasting performance than the choice of aggregating to daily or weekly time units. We had anticipated that with sparsity from relatively few cases in this outbreak, nowcasting at weekly aggregation might improve performance. This was not borne out, possibly because of greater difficulty in estimating the epidemic trend using fewer data points. Hindcasting was more accurate when counts were low and stable, toward the end of the outbreak. Others have also found that forecasting performance metrics varied between early and declining mpox outbreak phases [32]. This underscores the need for nowcasting methods that will reliably perform well as epidemics grow, peak, and decline.

Stratifying by race or ethnicity improved performance, and the highest average scores were observed for White patients. Performance at shorter windows was lowest for hindcasts of Hispanic or Latino patients, possibly due to a lower interview success rate.

### Limitations

Several data quality limitations were noted during project implementation. First, a quarter of diagnosed patients had missing onset dates, which made onset dates less reliable than diagnosis dates for monitoring trends. Patient interviews were the primary source for the onset date. Some patients may have refused interviews due to the sensitive nature of revealing a sexual history in the context of their mpox diagnosis. Generally, surveys about sexual history have participant refusal rates of 25%‐35% [35]. Another reason for missingness is that some patients could not recall their onset date.

As onset dates and race and ethnicity data were often collected during interviews, the stratified and onset-based nowcasts relied on incomplete reports (Table 3, Table S1 in Multimedia Appendix 1). Unstratified, diagnosis-based hindcasts were the only type of hindcast evaluated that relied only on complete and timely laboratory reporting data. Additionally, the median delay from onset to report decreased rapidly from the study start until late August (Figure S4 in Multimedia Appendix 1). Shortening delay distributions could have led NobBS to overestimate onsets in August. Shorter moving windows started with input data from the peak and early decline of the outbreak, while delay distributions and epidemic trends were rapidly changing. Longer moving windows, which appeared to be associated with better average scores, only began once case counts had stabilized, limiting our ability to directly compare window lengths.

Additionally, we included both confirmed and probable cases. Delays for both diagnosis to diagnosis report and onset to onset report were slightly shorter for confirmed than probable cases. While differences in delays by case status were minor, accounting for case status might improve accuracy. Additionally, stratified estimates were limited to Black or African American, Hispanic or Latino, and White patients, while unstratified estimates were for all patients, regardless of race or ethnicity, reducing our ability to directly compare stratified and unstratified estimates.

Although NobBS accounts for reporting delays, it does not account for other limitations of reportable disease data, including underascertainment, underreporting, and misdiagnosis or misclassification [19]. NobBS also does not account for external determinants influencing epidemic trends, such as behavioral changes or public health interventions. Our study period began after commercial laboratory testing became available, which nearly coincided with the epidemic peak, so we were unable to evaluate nowcasting performance during initial epidemic growth. We observed trade-offs in evaluation metrics, for example, scenarios of improved PI coverage with decreased accuracy (Table 5, Tables S4 and S5 in Multimedia Appendix 1), which could be related to overfit models or overconfident PIs. Additionally, the maximum delay used in NobBS of the moving window length minus 1 meant that window lengths were longer than the 90th percentile of observed delays for almost all moving windows. This could explain why changing the window lengths did not have a major impact on performance. Also, lags from diagnosis to report were almost universally less than 1 week, and nowcasting at weekly resolution may not be warranted for such short reporting delays. We did not compare NobBS with other nowcasting methods, such as generalized additive models [34,36], nor did we assess methods developed for the purpose of estimating the time-varying effective reproduction number instead of observed case counts [31].

### Practice Implications

**Figure 3.**
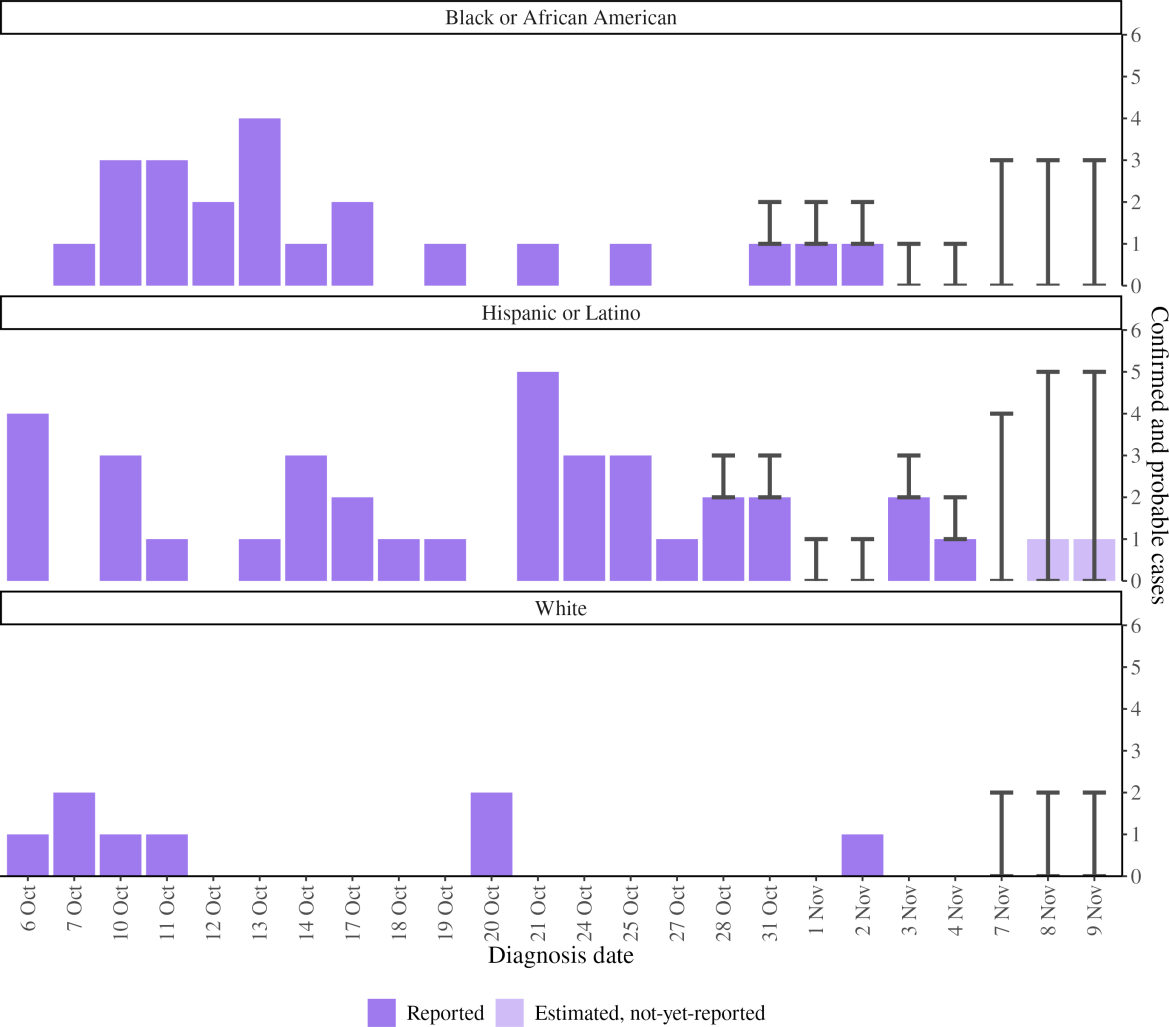
Hindcast visualization of reported and estimated (not-yet-reported) mpox cases diagnosed among New York City residents, presented to Incident Command System leadership on November 10, 2022. The error bars represent 95% prediction intervals.

**Figure 4.**
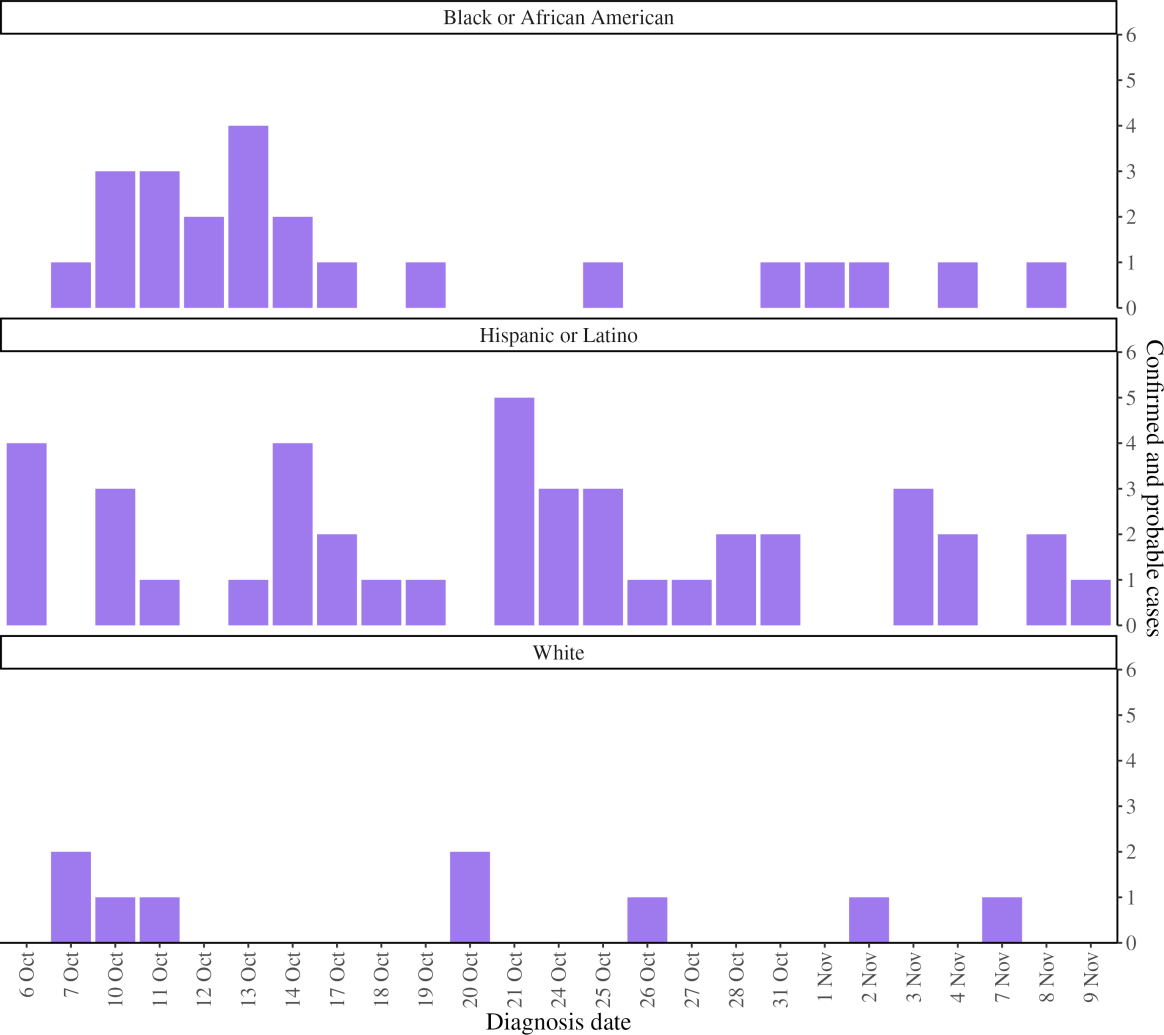
Mpox cases diagnosed among New York City residents for the same period as Figure 3, after data fully accrued.

Accurate nowcasts can facilitate real-time trend monitoring and reporting to policymakers. Stratifying nowcasts by key demographic characteristics associated with inequities, including disaggregated race or ethnicity groups, can help public health authorities quickly identify and remediate inequities faster than monitoring epidemic curves, without accounting for data lags. For example, on November 10, 2022, in the context of declining overall case counts and a focus on ensuring equitable access to interventions, we presented stratified nowcasting results to the Incident Command System leadership, highlighting that the number of recent estimated cases, even with uncertainty, was disproportionately higher among Hispanic or Latino New Yorkers (Figure 3). This finding was borne out after data fully accrued (Figure 4); final daily case counts were within the narrow 95% PIs for estimated case counts.

We recommend stratifying nowcasts to monitor differences in epidemic trends across patient groups and to improve performance, as well as using diagnosis date rather than onset date. For future outbreaks, health departments can strengthen preparedness to rapidly initiate nowcasting during public health emergencies by populating a field for onset report date directly in the surveillance database. Imputing the onset date might be necessary to improve completeness [31].

Performance metrics were sensitive to NobBS implementation details, and no single moving window length emerged as best performing. Health departments need reliable tools to initiate daily nowcasting by diagnosis date within the first few weeks of a public health emergency, to conduct interim performance evaluations to assess accuracy, and to pinpoint which adjustments to make to improve performance while emergencies are ongoing. Tools such as the scoringutils R package [37] could facilitate rapid evaluations and adjustments. Additional practical guidance is needed for health departments on how to optimize nowcasting, including how to add robustness by using multiple distinct methods, and how to best evaluate performance.

## Supplementary material

10.2196/56495 Multimedia Appendix 1 Additional details about patient characteristics by interview status, data lags, assessment of overdispersion in case counts, and Nowcasting by Bayesian Smoothing performance metrics by onset date and at weekly resolution.

## Abbreviations

MAE: mean absolute error
NobBS: Nowcasting by Bayesian Smoothing
PI: prediction interval
rRMSE: relative root mean square error
