# Supplementary material for: Nowcasting to Monitor Real-Time Mpox Trends During the 2022 Outbreak in New York City: Evaluation Using Reportable Disease Data Stratified by Race or Ethnicity
Source: Online J Public Health Inform. 2025 Jan 14;17:e56495. doi: 10.2196/56495 (PMC11750114; doi:10.2196/56495)
Supplement: Multimedia Appendix 1 [file ojphi-v17-e56495-s001.docx]

**Multimedia Appendix 1 (Supplemental Table and Figures)**

**Nowcasting to Monitor Real-Time Mpox Trends During the 2022 Outbreak in New York City: An Evaluation Using Reportable Disease Data Stratified by Race or Ethnicity**

Table S1. New York City residents diagnosed with mpox from July 8 through September 30, 2022, by interview status, race or ethnicity, and onset date availability.

| Variable | Interviewed (n=2429),  n (column %) | Not interviewed (n=876),  n (column %) | Total (n=3305),  n (column %) |
| --- | --- | --- | --- |
|  | | | |
| **Race or ethnicity** |  | |  |
| Asian or Pacific Islander | 91 (3.7) | 18 (2.1) | 109 (3.3) |
| Black or African American | 683 (28.1) | 236 (26.9) | 919 (27.8) |
| Hispanic or Latino | 943 (38.8) | 188 (21.5) | 1131 (34.2) |
| Other | 44 (1.8) | 12 (1.4) | 56 (1.7) |
| Unknown | 126 (5.2) | 248 (28.3) | 374 (11.3) |
| White | 542 (22.3) | 174 (19.9) | 716 (21.7) |
| **Onset date available** | | | |
| Yes | 2384 (98.1) | 109 (12.4) | 2493 (75.4) |
| No | 45 (1.9) | 767 (87.6) | 812 (24.6) |

Table S2. Lags from onset to diagnosis among New York City residents with mpox onset from July 8 through September 30, 2022.

| Onset period | Stratification | Median number of days from onset date to diagnosis date (IQR), 90^th^ percentile | No. of cases^a^ |
| --- | --- | --- | --- |
|  |  |  |  |
| July 8–Sep 30 | Unstratified | 4 (2–7), 10 | 2225 |
|  | Black or African American | 4 (2–7), 10 | 629 |
|  | Hispanic or Latino | 4 (2–7), 10 | 857 |
|  | White | 3 (2–6), 9 | 494 |
| July 8–31 | Unstratified | 4 (2–7), 11 | 1169 |
|  | Black or African American | 4 (3–7), 11 | 316 |
|  | Hispanic or Latino | 4 (2–7), 12 | 409 |
|  | White | 4 (2–6), 10 | 310 |
| August 1–31 | Unstratified | 3 (1–6), 9 | 832 |
|  | Black or African American | 3 (1–6), 9 | 245 |
|  | Hispanic or Latino | 4 (2–6), 9 | 348 |
|  | White | 3 (2–6), 8 | 152 |
| September 1–30 | Unstratified | 4 (2–6), 9 | 224 |
|  | Black or African American | 4 (2–6), 9 | 68 |
|  | Hispanic or Latino | 4 (3–7), 10 | 100 |
|  | White | 4 (2–6), 10 | 32 |

^a^Excludes 53 patients for whom diagnosis purportedly preceded illness onset.

Table S3. Median dispersion ratio from Poisson regression models of mpox cases diagnosed among New York City residents from July 13 through September 27, 2022, by diagnosis date and by onset date for period lengths used in real time.

| Date of interest | Period length^a^ | Dispersion ratio | *P*-value |
| --- | --- | --- | --- |
|  |  |  |  |
| Diagnosis | Full study period | 8.21 | <.001 |
|  | 14-day | 15.98^b^ | <.001 in all periods |
| Onset | Full study period | 2.67 | <.001 |
|  | 21-day | 2.30^b^ | <.05 in a plurality of periods^c^ |

^a^The full study period was evaluated, as well as a sequence of individual smaller periods representing scenarios used in real time for onset and diagnosis. For example, for 14-day periods, counts were summarized by date across each of the 14-day periods ending Tuesdays from July 26 through September 27, 2022, dispersion ratios and *P*-values were calculated for each period, and then the median dispersion ratio across periods was calculated.

^b^Median dispersion ratio across periods.

^c^Onset counts were less overdispersed starting with the 21-day period ending August 30, 2022.

Table S4. Performance measures for onset date-based hindcasting approaches in Nowcasting by Bayesian Smoothing (NobBS), applied to daily case counts of New York City residents with mpox onset from July 13 through September 27, 2022. Metrics calculated on last 7 days of hindcast.

| Scenario number | Stratification | Window length in days^a^ | Mean absolute error | Relative root mean square error^b^ | Number of estimates when the 95% prediction interval included the final case count (95% prediction interval coverage) | No. of estimates evaluated (No. of models run) | Average score |
| --- | --- | --- | --- | --- | --- | --- | --- |
|  |  |  |  |  |  |  |  |
| 1 | Unstratified | 14 | 8.10 | 0.75 | 69 (98.57) | 70 (10) | 0.30 |
| 2^c^ |  | 21 | 12.48 | 1.07 | 53 (84.13) | 63 (9) | 0.23 |
| 3 |  | 28 | 10.63 | 1.12 | 42 (75.00) | 56 (8) | 0.20 |
| 4 |  | 35 | 10.53 | 1.26 | 37 (75.51) | 49 (7) | 0.18 |
| 5 |  | 42 | 10.24 | 1.42 | 34 (80.95) | 42 (6) | 0.16 |
| 6 |  | 49 | 6.97 | 1.31 | 30 (85.71) | 35 (5) | 0.26 |
| 7 | Black or African American | 14 | 2.63 | 0.69 | 68 (97.14) | 70 (10) | 0.40 |
| 8^c^ |  | 21 | 2.89 | 0.90 | 59 (93.65) | 63 (9) | 0.40 |
| 9 |  | 28 | 2.70 | 1.17 | 54 (96.43) | 56 (8) | 0.42 |
| 10 |  | 35 | 3.04 | 1.41 | 46 (93.88) | 49 (7) | 0.41 |
| 11 |  | 42 | 2.71 | 1.41 | 38 (90.48) | 42 (6) | 0.45 |
| 12 |  | 49 | 1.80 | 1.62 | 34 (97.14) | 35 (5) | 0.55 |
| 13 | Hispanic or Latino | 14 | 3.89 | 1.11 | 66 (94.29) | 70 (10) | 0.25 |
| 14^c^ |  | 21 | 4.11 | 1.33 | 59 (93.65) | 63 (9) | 0.26 |
| 15 |  | 28 | 3.96 | 1.51 | 51 (91.07) | 56 (8) | 0.26 |
| 16 |  | 35 | 4.04 | 1.69 | 43 (87.76) | 49 (7) | 0.24 |
| 17 |  | 42 | 4.12 | 2.03 | 34 (80.95) | 42 (6) | 0.24 |
| 18 |  | 49 | 2.57 | 1.14 | 33 (94.29) | 35 (5) | 0.38 |
| 19 | White | 14 | 2.03 | 0.88 | 69 (98.57) | 70 (10) | 0.50 |
| 20^c^ |  | 21 | 2.83 | 1.39 | 61 (96.83) | 63 (9) | 0.43 |
| 21 |  | 28 | 2.63 | 1.56 | 51 (91.07) | 56 (8) | 0.42 |
| 22 |  | 35 | 2.14 | 1.71 | 45 (91.84) | 49 (7) | 0.49 |
| 23 |  | 42 | 1.76 | 1.62 | 40 (95.24) | 42 (6) | 0.61 |
| 24 |  | 49 | 1.37 | 1.12 | 34 (97.14) | 35 (5) | 0.74 |
| 25 | All Stratified | 14 | 2.85 | 0.91 | 203 (96.67) | 210 (10) | 0.37 |
| 26^c^ |  | 21 | 3.28 | 1.22 | 179 (94.71) | 189 (9) | 0.36 |
| 27 |  | 28 | 3.10 | 1.42 | 156 (92.86) | 168 (8) | 0.36 |
| 28 |  | 35 | 3.07 | 1.61 | 134 (91.16) | 147 (7) | 0.36 |
| 29 |  | 42 | 2.87 | 1.71 | 112 (88.89) | 126 (6) | 0.40 |
| 30 |  | 49 | 1.91 | 1.32 | 101 (96.19) | 105 (5) | 0.54 |

^a^14-, 21-, 28-, 35-, 42-, and 49-day nowcasts started on July 26, August 2, August 9, August 16, August 23, and August 30, 2022, respectively, to provide 2, 3, 4, 5, 6, or 7 weeks of Wednesday–Tuesday data since study start date July 8, 2022. We mimicked nowcasts weekly, ending September 27, 2022, as the last Tuesday during the study period.

^b^There were 13 cells in which the final count of onsets stratified by race or ethnicity was 0. These observations were excluded from the calculation of rRMSE.

^c^Indicates scenario applied in real-time at the New York City Health Department.

Figure S1. Comparison of 7-day hindcasts conducted on Wednesdays using various moving window lengths at the weekly time unit for confirmed and probable mpox cases among New York City residents diagnosed from July 19 through September 27, 2022, overall and stratified by three race or ethnicity groups. Final case counts reported as of September 1, 2023, are shown in black.


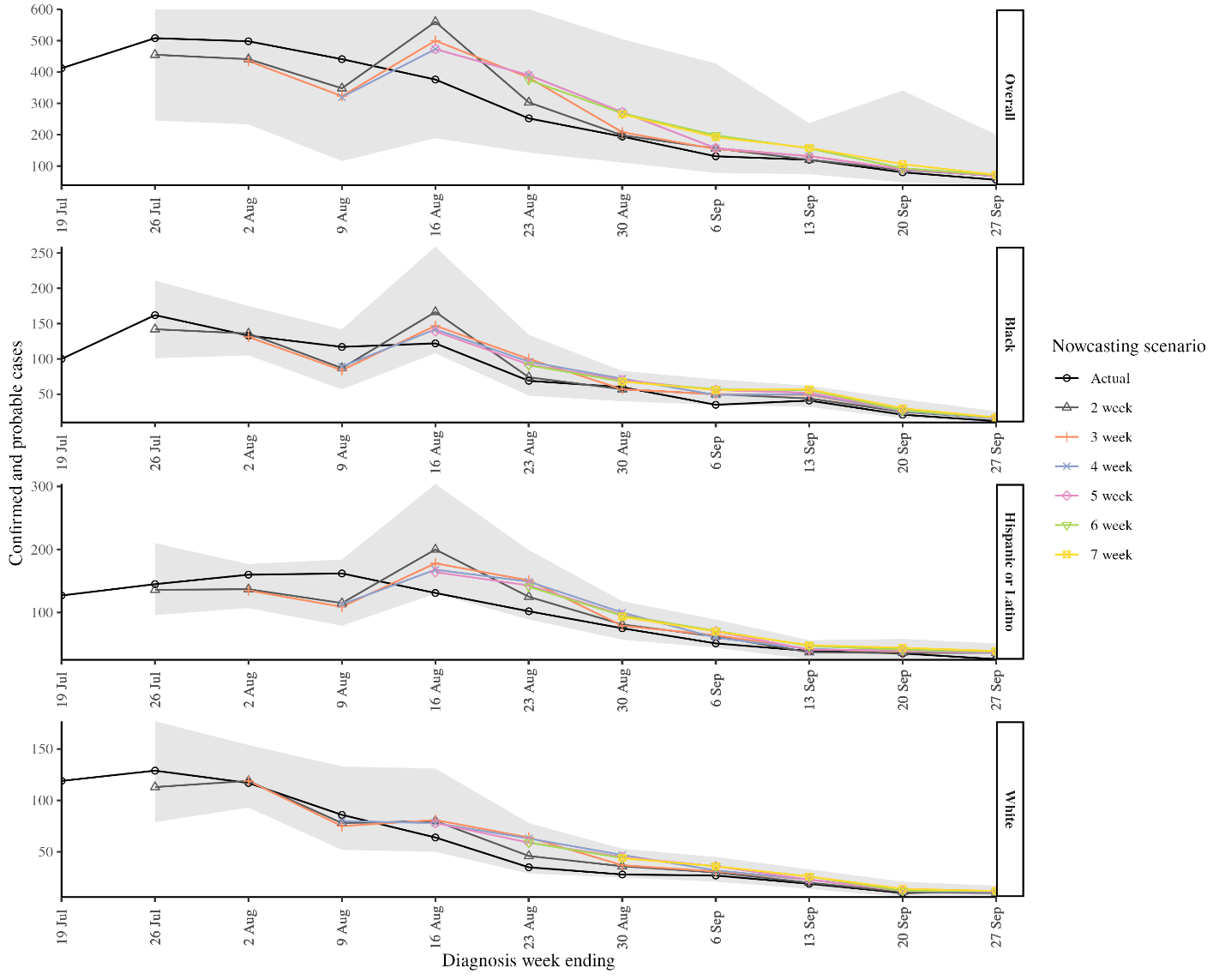


The 95% prediction interval is shown in grey for the 2-week window, for comparison with the 14-day scenario implemented in real time. The y-axis for the overall diagnosis plot was truncated at 600 for clarity, but the observed upper bound of the 95% prediction interval for the 2-week window was 28,977 on August 9, 2022.

Figure S2. Comparison of 7-day hindcasts conducted on Wednesdays using various moving window lengths at the daily time unit for confirmed and probable mpox cases among NYC residents with onsets from July 8 through September 27, 2022, overall and stratified by three race or ethnicity groups. Final onset counts reported as of September 1, 2023, are shown in black.


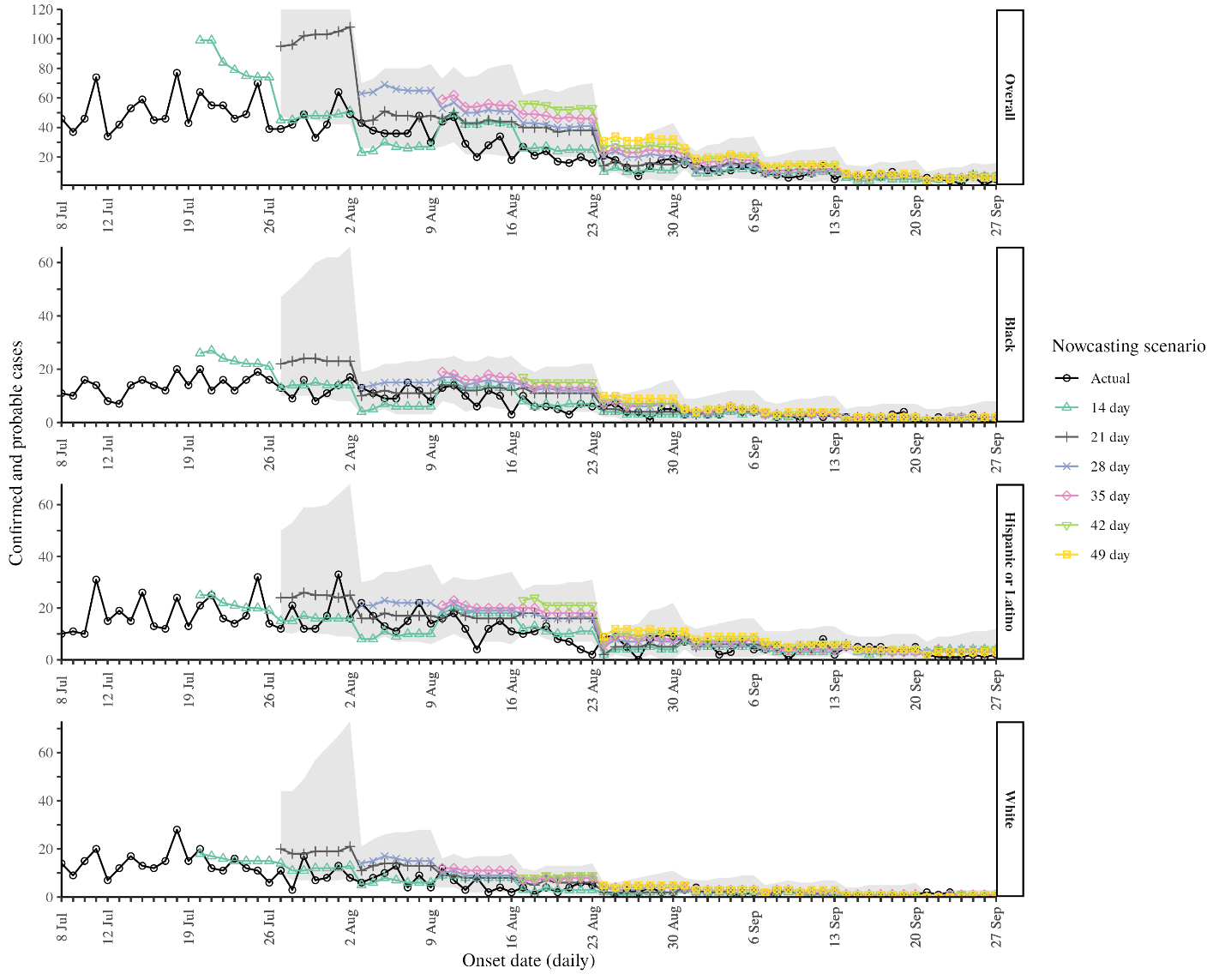


The 95% prediction interval is shown in grey for the 21-day window, which was the scenario implemented in real time. The y-axis for overall onsets was truncated at 120 for clarity, but the observed upper bound of the 95% prediction interval for the 21-day window was 673 on August 2, 2022.

Figure S3. Comparison of 7-day hindcasts conducted on Wednesdays using various moving window lengths at the weekly time unit for confirmed and probable mpox cases among NYC residents with onsets from July 19 through September 27, 2022, overall and stratified by three race or ethnicity groups. Final onset counts reported as of September 1, 2023, are shown in black.


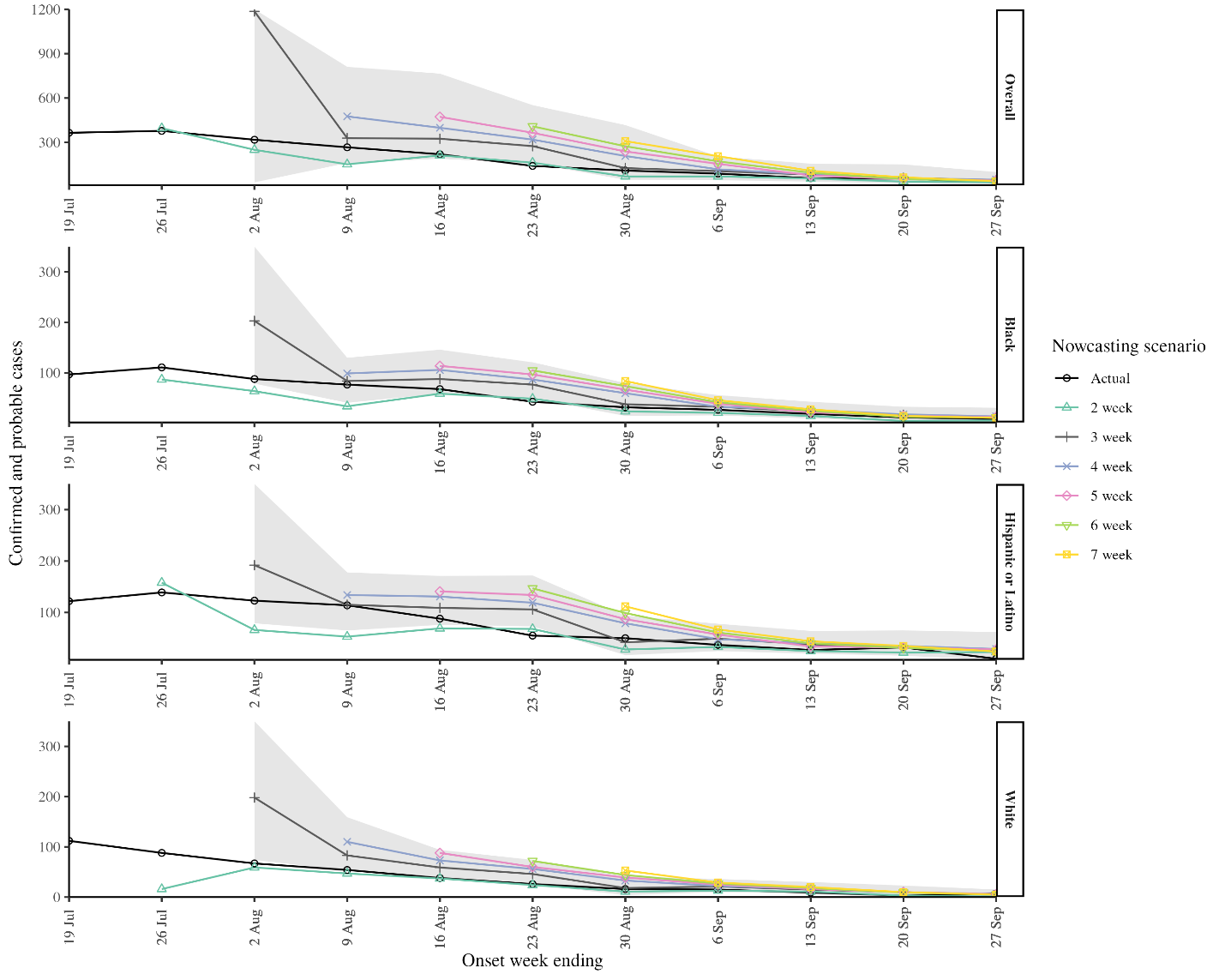


The 95% prediction interval is shown for the 3-week window, for comparison with the 21-day scenario implemented in real time. The y-axes for the overall and stratified onset plots were truncated at 1200 and 350 respectively for clarity, but the observed upper bound of the 95% prediction interval for the 3-week window was 40,250 in the overall plot and 765 in a stratified plot, both on August 2, 2022.

Figure S4. Median weekly delay from onset to onset report and from diagnosis to diagnosis report among New York City residents with confirmed or probable mpox from July 8 through September 30, 2022.


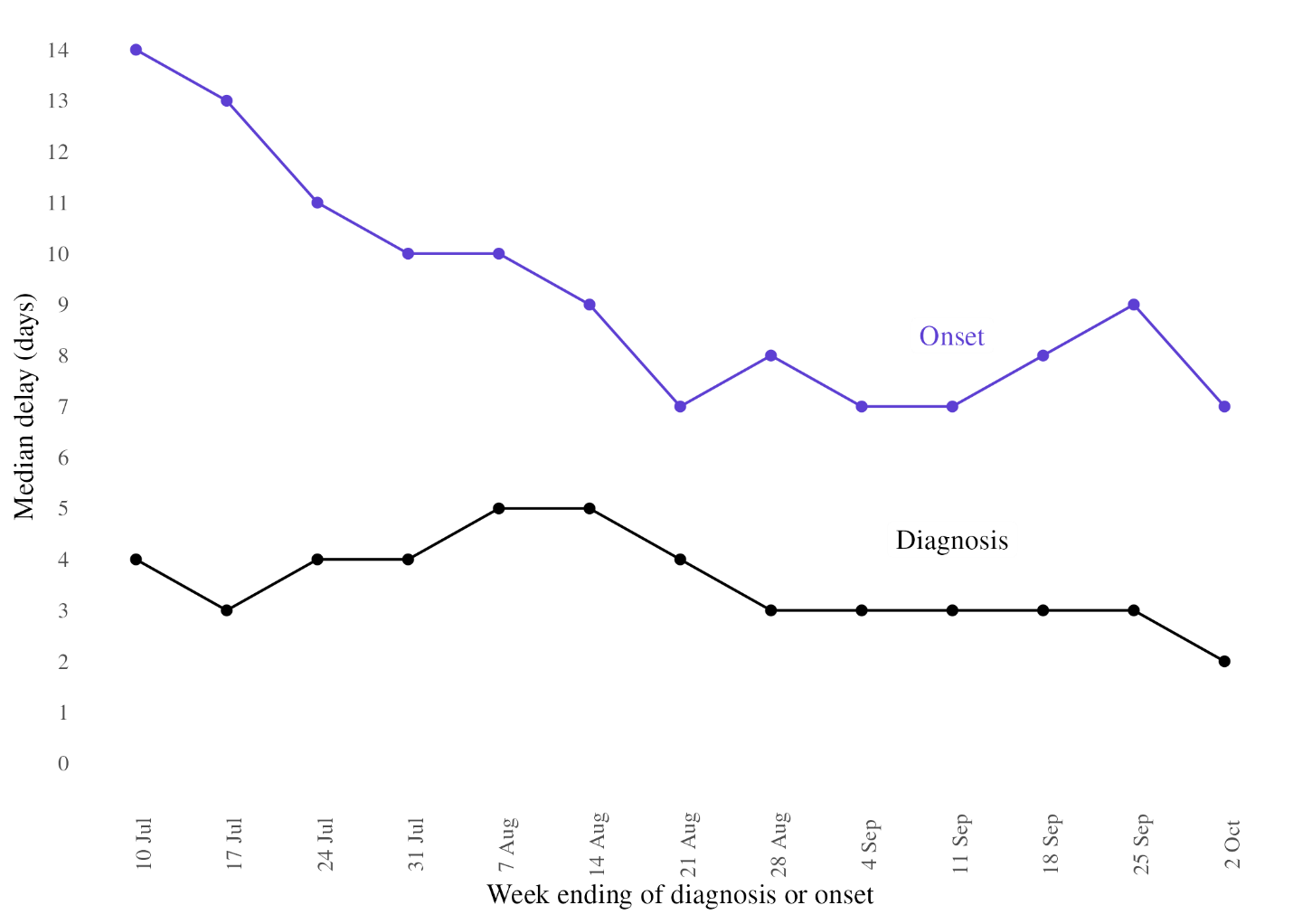


Table S5. Performance measures for weekly^a^ hindcasting approaches in Nowcasting by Bayesian Smoothing (NobBS), applied to case counts of New York City residents with mpox diagnosis or onset from July 13 through September 27, 2022. Metrics calculated on last week of hindcast.

| Scenario number | Date of interest | Stratification | Window length in weeks^b^ | Mean absolute error | Relative root mean square error | Number of estimates when the 95% prediction interval included the final case count (95% prediction interval coverage) | Number of estimates evaluated (number of models run) | Average score |
| --- | --- | --- | --- | --- | --- | --- | --- | --- |
|  |  |  |  |  |  |  |  |  |
| 1 | Diagnosis | Unstratified | 2 | 48.70 | 0.21 | 10 (100.00) | 10 (10) | 0.27 |
| 2 |  |  | 3 | 56.33 | 0.25 | 9 (100.00) | 9 (9) | 0.29 |
| 3 |  |  | 4 | 61.63 | 0.30 | 7 (87.50) | 8 (8) | 0.25 |
| 4 |  |  | 5 | 53.14 | 0.30 | 6 (85.71) | 7 (7) | 0.30 |
| 5 |  |  | 6 | 54.67 | 0.37 | 5 (83.33) | 6 (6) | 0.27 |
| 6 |  |  | 7 | 42.40 | 0.36 | 5 (100.00) | 5 (5) | 0.29 |
| 7 |  | Black or African American | 2 | 13.15 | 0.24 | 10 (100.00) | 10 (10) | 0.32 |
| 8 |  |  | 3 | 14.11 | 0.29 | 8 (88.89) | 9 (9) | 0.32 |
| 9 |  |  | 4 | 14.88 | 0.29 | 7 (87.50) | 8 (8) | 0.35 |
| 10 |  |  | 5 | 13.29 | 0.35 | 7 (100.00) | 7 (7) | 0.39 |
| 11 |  |  | 6 | 13.00 | 0.39 | 5 (83.33) | 6 (6) | 0.35 |
| 12 |  |  | 7 | 11.80 | 0.42 | 4 (80.00) | 5 (5) | 0.36 |
| 13 |  | Hispanic or Latino | 2 | 20.10 | 0.25 | 10 (100.00) | 10 (10) | 0.23 |
| 14 |  |  | 3 | 23.00 | 0.28 | 8 (88.89) | 9 (9) | 0.16 |
| 15 |  |  | 4 | 23.00 | 0.29 | 5 (62.50) | 8 (8) | 0.19 |
| 16 |  |  | 5 | 18.86 | 0.30 | 5 (71.43) | 7 (7) | 0.26 |
| 17 |  |  | 6 | 17.50 | 0.33 | 4 (66.67) | 6 (6) | 0.26 |
| 18 |  |  | 7 | 13.60 | 0.34 | 4 (80.00) | 5 (5) | 0.26 |
| 19 |  | White | 2 | 6.80 | 0.18 | 10 (100.00) | 10 (10) | 0.49 |
| 20 |  |  | 3 | 8.78 | 0.33 | 8 (88.89) | 9 (9) | 0.39 |
| 21 |  |  | 4 | 9.88 | 0.40 | 7 (87.50) | 8 (8) | 0.35 |
| 22 |  |  | 5 | 10.00 | 0.39 | 5 (71.43) | 7 (7) | 0.35 |
| 23 |  |  | 6 | 9.67 | 0.42 | 4 (66.67) | 6 (6) | 0.32 |
| 24 |  |  | 7 | 7.20 | 0.38 | 4 (80.00) | 5 (5) | 0.46 |
| 25 |  | All Stratified | 2 | 13.35 | 0.23 | 30 (100.00) | 30 (10) | 0.33 |
| 26 |  |  | 3 | 15.30 | 0.30 | 24 (88.89) | 27 (9) | 0.27 |
| 27 |  |  | 4 | 15.92 | 0.33 | 19 (79.17) | 24 (8) | 0.29 |
| 28 |  |  | 5 | 14.05 | 0.35 | 17 (80.95) | 21 (7) | 0.33 |
| 29 |  |  | 6 | 13.39 | 0.38 | 13 (72.22) | 18 (6) | 0.31 |
| 30 |  |  | 7 | 10.87 | 0.38 | 12 (80.00) | 15 (5) | 0.35 |
| 31 | Onset | Unstratified | 2 | 31.20 | 0.24 | 10 (100.00) | 10 (10) | 0.15 |
| 32 |  |  | 3 | 138.56 | 1.01 | 8 (88.89) | 9 (9) | 0.18 |
| 33 |  |  | 4 | 92.13 | 0.74 | 6 (75.00) | 8 (8) | 0.11 |
| 34 |  |  | 5 | 101.00 | 0.93 | 5 (71.43) | 7 (7) | 0.09 |
| 35 |  |  | 6 | 94.00 | 1.10 | 4 (66.67) | 6 (6) | 0.11 |
| 36 |  |  | 7 | 76.80 | 1.08 | 3 (60.00) | 5 (5) | 0.17 |
| 37 |  | Black or African American | 2 | 13.40 | 0.33 | 10 (100.00) | 10 (10) | 0.29 |
| 38 |  |  | 3 | 22.44 | 0.57 | 8 (88.89) | 9 (9) | 0.30 |
| 39 |  |  | 4 | 19.63 | 0.61 | 6 (75.00) | 8 (8) | 0.17 |
| 40 |  |  | 5 | 22.43 | 0.73 | 4 (57.14) | 7 (7) | 0.16 |
| 41 |  |  | 6 | 21.50 | 0.85 | 4 (66.67) | 6 (6) | 0.18 |
| 42 |  |  | 7 | 17.00 | 0.83 | 4 (80.00) | 5 (5) | 0.23 |
| 43 |  | Hispanic or Latino | 2 | 22.00 | 0.51 | 10 (100.00) | 10 (10) | 0.15 |
| 44 |  |  | 3 | 21.11 | 0.70 | 8 (88.89) | 9 (9) | 0.17 |
| 45 |  |  | 4 | 25.25 | 0.86 | 6 (75.00) | 8 (8) | 0.08 |
| 46 |  |  | 5 | 30.57 | 0.97 | 5 (71.43) | 7 (7) | 0.07 |
| 47 |  |  | 6 | 32.33 | 1.05 | 5 (83.33) | 6 (6) | 0.08 |
| 48 |  |  | 7 | 25.20 | 0.96 | 4 (80.00) | 5 (5) | 0.17 |
| 49 |  | White | 2 | 10.50 | 0.40 | 10 (100.00) | 10 (10) | 0.46 |
| 50 |  |  | 3 | 24.89 | 1.11 | 8 (88.89) | 9 (9) | 0.25 |
| 51 |  |  | 4 | 20.13 | 1.16 | 5 (62.50) | 8 (8) | 0.14 |
| 52 |  |  | 5 | 18.86 | 1.31 | 4 (57.14) | 7 (7) | 0.14 |
| 53 |  |  | 6 | 17.17 | 1.41 | 4 (66.67) | 6 (6) | 0.21 |
| 54 |  |  | 7 | 14.20 | 1.63 | 4 (80.00) | 5 (5) | 0.25 |
| 55 |  | All Stratified | 2 | 15.30 | 0.42 | 30 (100.00) | 30 (10) | 0.27 |
| 56 |  |  | 3 | 22.81 | 0.83 | 24 (88.89) | 27 (9) | 0.24 |
| 57 |  |  | 4 | 21.67 | 0.91 | 17 (70.83) | 24 (8) | 0.12 |
| 58 |  |  | 5 | 23.95 | 1.03 | 13 (61.90) | 21 (7) | 0.12 |
| 59 |  |  | 6 | 23.67 | 1.13 | 13 (72.22) | 18 (6) | 0.14 |
| 60 |  |  | 7 | 18.80 | 1.19 | 12 (80.00) | 15 (5) | 0.21 |

^a^Weekly analyses were conducted on Wednesdays, aggregating data into 7-day periods ending the prior day (ie, Wednesday–Tuesday).

^b^2-, 3-, 4-, 5-, 6-, and 7-week nowcasts started on July 26, August 2, August 9, August 16, August 23, and August 30, 2022, respectively, to provide 2, 3, 4, 5, 6, or 7 weeks of Wednesday–Tuesday data since study start date on July 8, 2022. All weekly nowcasts ended on September 27, 2022, the last Tuesday in the window.
